# Gap enhanced fluorescence as a road map for the detection of very weakly fluorescent emitters from visible to ultraviolet

**DOI:** 10.1038/s41598-017-14250-x

**Published:** 2017-10-27

**Authors:** Duncan McArthur, Francesco Papoff

**Affiliations:** Department of Physics, SUPA, University of Strathclyde, 107 Rottenrow, Glasgow, G4 0NG UK

## Abstract

We analyze the enhancement of the rates of both the emission and the far field radiation for dipoles placed in the gap between a metallic nanorod, or nanosphere, and a metallic substrate. For wavelengths between 150 nm and 650 nm, the response of the gapped nanostructures considered in this work is dominated by few principal modes of the nanoparticle, which include self-consistently the effect of the substrate. For wavelengths shorter than 370 nm, the far field radiative enhancements of aluminum nanostructures are significantly higher than those for gold or silver. With aluminum, bright mode resonances are tunable over tens or hundreds of nanometers by changing the size of the nanoparticle and have far field radiative enhancements of up to three orders of magnitude. These results provide a road map to label-free detection of many emitters too weakly fluorescent for present approaches.

## Introduction

Nanostructures can induce large variations in many fundamental quantum phenomena such as the rate of spontaneous emission^1,2^, the photonic Lamb shift of resonance frequencies and Casimir-Polder forces^3^. These effects have been extensively investigated with noble metal nanoparticles^4,5^ in the visible range. Observing these effects in the ultraviolet is not trivial because the electromagnetic response of most materials depends strongly on the frequency of light, so experimental setups effective in one part of the electromagnetic spectrum cannot be directly reproduced in another. Here we consider how metal nanoparticles and substrates can enhance, by orders of magnitude, the detection of many important molecules that have radiative decays in the ultraviolet much weaker than nonradiative decays. Examples of such molecules are alkanes^6^, most amino acids^7^ in proteins and peptides, and DNA bases.

We consider both the enhancement of the emission rate, *i.e*. the rate of decay events in which a photon is emitted, and of the far field radiative rate, *i.e*. the rate of decay events leading to the emission of photons propagating in the far field. The emission rate affects the lifetime of the emitter, and the far field emission rate is proportional to the fraction of decay events detected in the far field, where fluorescence signals are detected in most experiments.

We investigate nanospheres and nanorods comprised of gold, silver or aluminum held above a substrate made of the same material as the nanoparticle. The interest in aluminum in nanophotonics is relatively more recent than for gold and silver, and it is due to aluminum being abundant, low cost and with plasmon modes in the visible and the ultraviolet^8–13^. We find that aluminum nanoparticles and substrates are ideally suited for label-free detection of weakly fluorescent molecules in the ultraviolet because they have resonances with a much stronger far field radiative enhancement than similar nanostructures of gold or silver for wavelengths shorter than 370 nm. These resonances are tunable between 150 nm and 650 nm and can produce a simultaneous enhancement of the decay rates of dipolar emitters placed in the gap between the nanoparticle and the substrate, and of the radiation emitted in the far field. Compared to nanospheres, nanorods have sharper resonances and stronger far field enhancements.

The rest of the paper is organized as follows. First we give a description of the systems considered, then we give an outline of the theory, the main numerical results and the conclusions. A more technical summary of the theory can be found in the methods section.

## Results and Discussion

### Practical considerations

**Figure 1:**
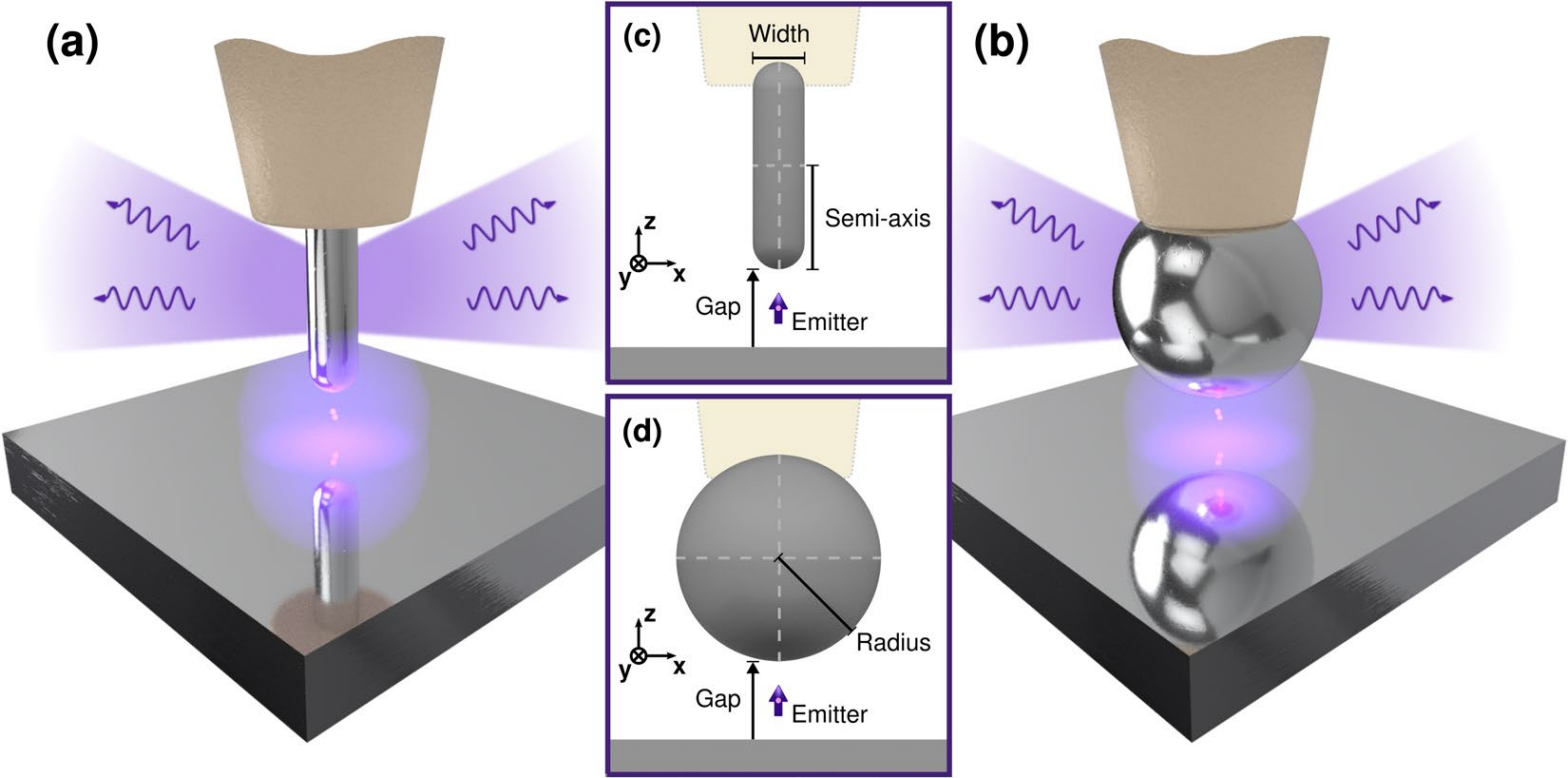
Simple schematic of a metallic nanorod (**A**) and a nanosphere (**B**) coupled to substrate made of the same metal. The nanoparticle is held into position by a dielectric cantilever. The range of angles with the strongest emission of the far field radiation is shown in light blue and it is symmetric about the vertical axis. Geometrical parameters for the rod, (**C**) and the sphere, (**D**), not to scale. The dipole is held in place by a dielectric spacer (not shown).

A metallic nanostructure can significantly enhance the detection of fluorescence by emitters in which the emission rate is much less than the internal nonradiative decay rate^14^, if it enhances the far field radiation rate of the emitter. This happens when the emitter is strongly coupled to electromagnetic modes that efficiently transport energy in the far field, but not when the emitter is coupled mainly to surface modes that are confined close to the metallic nanostructure. We are interested in finding nanostructures able to enhance far field radiative rates and we consider nanospheres and nanorods with their axis orthogonal to the substrate and gaps varying from 5 nm to 20 nm, see Fig. 1, typical of Scanning Near field Microscopy^15^ and patch antennas^1^. We assume that the dipole emitter is held in place in the middle of the gap between the substrate and the nanoparticle by a dielectric spacer - not shown - which inhibits charge transfer between the emitter and the substrate. A small patch of SiO_2_ would be a suitable spacer for most wavelengths considered here, but other solutions have been used^5,16^.

From a practical point of view, note that tilts of the axis of the nanorod break the rotational symmetry and induce mixing among modes with different azimuthal index, *i.e*. modes that transform differently under rotation. However, these effects are weak and can be neglected for nanorods with the width we consider and for tilting angles of a few degrees. Modes with higher azimuthal index may affect also the response of particles that are not perfectly spherical; however, these modes do not have strong resonances in the range of wavelengths considered, and so these effects are also weak. As long as the dielectric tip does not interfere with the radiation patterns, its effect should be limited to a small shift of the resonant wavelengths. From the radiation patterns shown in the following, we see that this is the case for the brightest resonances, hence we neglect the presence of the tip throughout.

### Theory

In classical electromagnetism, the electric field measured at a point *x*, generated by an electric dipole positioned at *x*′, is given by $E(x)={G}^{EP}(x,x^{\prime} ){\bf{p}}(x^{\prime} )$, where the 3 × 3 operator ${G}^{EP}(x,x^{\prime} )$ is the electric dyadic Green’s function and the vector **p** is the dipole moment. It can be shown that the Green’s function also plays a crucial role in quantum optics^17^, giving the electromagnetic fields generated by quantum fluctuations in polarization and magnetization^18–21^. In particular, in the usual weak coupling limit^17^ the emission rate of an electric dipole, radiating at frequency *ω*, depends on the imaginary part of the Green’s function,

1 $${\rm{\Gamma }}(\omega ;{\bf{p}})=\frac{2}{\hslash }{{\bf{p}}}^{{\rm{T}}}(x)Im[{G}^{EP}(\omega ;x,x)]{\bf{p}}(x),$$

where the products on the right-hand sides of Eq. (1) are standard vector-matrix products, $\hslash$ is the Plank constant divided by 2*π*, **p** ^T^ is the transpose of **p** and the field point and the position of the emitter coincide ($x=x^{\prime}$). The far field radiative rates are found using the dyadic Green’s function and the relation ${\rm{\Gamma }}/{{\rm{\Gamma }}}_{0}=P/{P}_{0}$, where ${{\rm{\Gamma }}}_{0}$ is the emission rate in vacuum, *P* is the total power emitted by the dipole in the presence of the nanostructure and *P*_0_ is the total power emitted by the dipole in vacuum^22^. The conservation of energy gives $P={P}_{r}+{P}_{abs}$, where *P*_*abs*_ is the energy absorbed by the metallic nanostructure and *P*_*r*_ is the energy radiated above the substrate, which is evaluated by integrating the far field asymptotic form of the modulus squared of the electric field, given by the Green’s function, over the upper semi-space.

When both *x* and *x*′ are located outside the nanoparticle and above the substrate, the Green’s function is given by the sum of two terms, ${G}^{EP}={G}_{0}^{EP}+{G}^{EP;S}$. The first term, ${G}_{0}^{EP}$, gives the sum of the field emitted by the dipole, which is known analytically, and the field reflected by the substrate, which is calculated using a continuous spectrum of propagating and evanescent plane waves^23,24^. The main difficulties in evaluating Eq. (1) comes from the second term, ${G}^{EP;S}$, the scattering Green’s function, which propagates the field from the dipole to the nanoparticle and then to the field point, taking into account multiple scattering between the particle and the substrate. Green’s functions can be obtained via numerical solutions of volume integral equations or finite-difference time-domain calculations^25^ at a high computational cost, and without a clear indication of the nature of the main contributions underlying the response. Modal decompositions of the Green’s functions for simple particles give efficient approximation schemes with good calculation speeds and the ability to provide greater physical insight^26–28^.

Our analysis is based on the expansion of the scattering Green’s function in the principal scattering modes of the particle^29^,

2 $${G}^{EP;S}(x,x^{\prime} ){\bf{p}}(x^{\prime} )=\sum _{n}{a}_{n}^{s}({\bf{p}}(x^{\prime} )){S}_{n}^{E}(x)$$

where ${S}_{n}^{E}(x)$ is the electric field of the $n-th$ principal mode at the point *x*, and ${a}_{n}^{s}({\bf{p}}(x^{\prime} ))$ is the amplitude of ${S}_{n}^{E}$ due to the field generated by the dipole **p** in *x*′. These principal modes include multiple scattering at all orders between the particle and the substrate and are a generalization of the Mie modes of a sphere^30^, with internal and scattering principal modes correlated pairwise on the surface of the particle. The amplitudes, ${a}_{n}^{s}({\bf{p}}(x^{\prime} ))$, are the product of two terms: one is the “sensitivity” of the mode, which is an intrinsic property of the particle that depends only on the spatial correlation between the scattering mode and its corresponding internal mode, at the surface of the particle; the other is proportional to the spatial correlation, at the surface of the particle, between the field generated by the dipole and the scattering and internal modes, see methods. This approach^29^ can be applied to inhomogeneous host media as long as all media are causal^17^ and nonlocal effects^31,32^ and quantum spill-out can be neglected^33^.

Using the relation between ${\rm{\Gamma }}$ and *P* and Eq. (2), we can see that ${{\rm{\Gamma }}}_{r}$ is due to the coupling of the emitter with the principal modes that efficiently transport energy in the far field, a property which can be easily identified from their asymptotic far field expressions^30^. The optimal conditions for enhancing far field detection of fluorescence are thus provided by structures in which one radiative mode is resonant and strongly coupled with the emitter, as we see in the following section.

### Numerical results

In this section we use the dielectric functions for gold, silver and aluminum given in^34^ to examine which of these metals enables the largest enhancement of the far field radiative rate, particularly in the ultraviolet. We then investigate how peaks in the enhancements can be tuned in order to find the optimal structure, over wavelengths ranging from 150 nm to 650 nm, and how the ratio between emission rate and far field radiative rate changes with the size of the gap. By examining properties of the modes, we show how they explain the observed responses of the nanostructures considered. For the nanorods, we vary the length of the semi-axis but maintain a constant width of 30 nm in all instances, and employ a geometry with semi- spherical end caps, *i.e*. where the radius of the cap is equivalent to half the width of the cylindrical body of the particle. For both types of particle, emission and radiative rates are enhanced only for dipoles polarized along the normal to the substrate, ${\bf{p}}={p}_{z}\hat{{\bf{z}}}$, and by modes symmetric under rotations about the normal.

**Figure 2:**
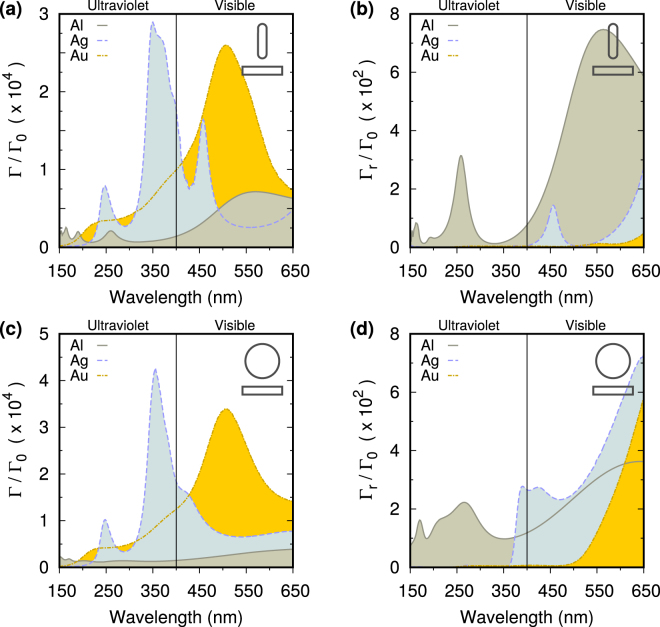
(**a**) Purcell factors (enhancement of the emission rate) ${\rm{\Gamma }}/{{\rm{\Gamma }}}_{0}$, for a dipole in the middle of a 5 nm gap and polarization orthogonal to the substrate, versus wavelength for nanorods with a semi-axis length of 60 nm and substrates both made of aluminum (grey solid), silver (blue dashed), and gold (yellow dot- dashed). (**b**) Far field radiative enhancement ${{\rm{\Gamma }}}_{r}/{{\rm{\Gamma }}}_{0}$ for the same nanostructures. (**c**) and (**d**) as in (**a**) and (**b**), respectively, but for nanospheres with 60 nm radius and a 5 nm gap. Gold and silver can have larger Purcell factors than aluminum across the considered spectrum, but for both particles only aluminum has significant radiative resonances in the deep ultraviolet.

In Fig. 2 we show the Purcell factors, the enhancement of the emission rate ${\rm{\Gamma }}/{{\rm{\Gamma }}}_{0}$, and the far field radiative enhancement, ${{\rm{\Gamma }}}_{r}/{{\rm{\Gamma }}}_{0}$, for a dipole in the middle of a 5 nm gap between a nanorod, or a nanosphere, and a substrate both made of aluminum, gold or silver. Strong radiative resonances depend on the shape of the particle, and can be observed for wavelengths longer than the plasmon resonance’s wavelength where the metal has a higher reflectivity. Gold and silver have plasmon resonances within the considered spectrum, while aluminum also has a strong plasmon resonance at shorter wavelengths. As a consequence, gold and silver can have larger Purcell factors than aluminum from 150 nm to 650 nm, but only aluminum has significant radiative resonances below 370 nm, as one would expect also by comparing the dielectric constants of these metals^13^. For this reason, we focus in the following on aluminum nanostructures.

**Figure 3:**
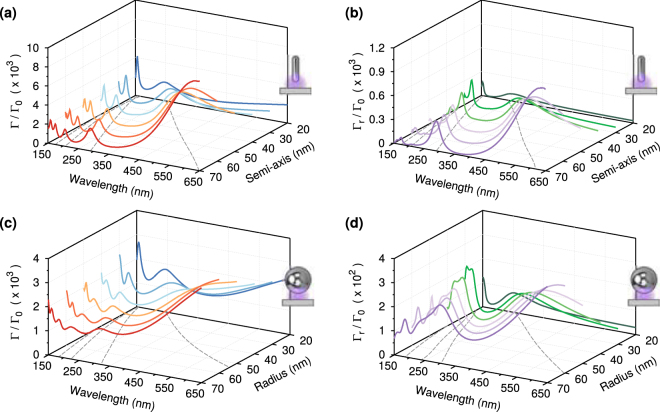
(**a**) Purcell factors in the middle of a 5 nm gap versus wavelength for nanorods with semi-axis varying between 20 nm and 70 nm in steps of 10 nm. The dashed lines are polynomial fits of the positions of the maxima. (**b**) Far field radiative enhancements ${{\rm{\Gamma }}}_{r}/{{\rm{\Gamma }}}_{0}$ for the same nanostructures as (**a**). The second broadest resonances have enhancements of the radiative decays of more than two orders of magnitude in the deep ultraviolet. Purcell factors (**c**) and far field radiative enhancements (**d**) of aluminum nanospheres with radii varying between 20 nm and 70 nm in steps of 10 nm, with a gap of 5 nm. The spectral features of the nanospheres are less sharp than those of the nanorods. All the resonances have far field radiative enhancements of more than two orders of magnitude in the deep ultraviolet.

We demonstrate the tunability of the resonances for aluminum nanostructures by varying the length of the semi-axis of the nanorods, and radius of the nanospheres, between 20–70 nm in increments of 10 nm while maintaining a gap of 5 nm. In Fig. 3(a) and (c), we compare the Purcell factors for these sets of nanorods and nanospheres, respectively. Similarly, in Fig. 3(b) and (d) we show the far field radiative enhancements for the same sets of structures. Both nanorods and nanospheres show peaks in which the Purcell factor at the dipole’s position is strongly enhanced, and which are blue-shifted as the length of the semi-axis, or radius, is decreased. The positions of these peaks primarily depend upon the length of the major axis for the nanorod, and the radius of the nanosphere, and are almost spectrally coincident for particles of equivalent length, but the peaks of the nanorods are narrower and more clearly resolved. By comparing the sets of results shown in Fig. 3, we can see that the two peaks at longer wavelengths in the Purcell factors correspond with large far field radiative enhancements for the nanorods. While for nanospheres, the peaks in the far field radiative enhancements are generally weaker and broader than the corresponding peaks for nanorods, with the second largest broad feature in the far field radiative enhancements being associated to two consecutive peaks in the Purcell factors.

**Figure 4:**
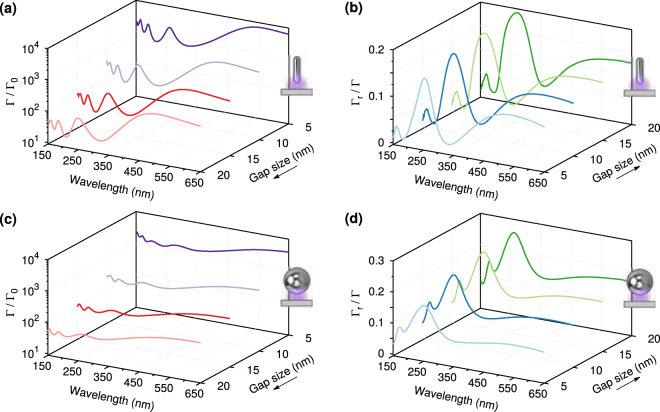
(**a**) Purcell factors for a nanorod with semi- axis of 60 nm and gaps of 5 nm, 10 nm, 15 nm and 20 nm. The increase in Purcell factor as the gap is reduced is of two orders of magnitude and it is larger for weakly radiative modes than for radiative modes. (**b**) The ratios ${{\rm{\Gamma }}}_{r}/{\rm{\Gamma }}$ for these nanostructures show only rises of a few percent as the gap is increased. (**c**) Purcell factors for a nanosphere with radius of 60 nm and gaps of 5 nm, 10 nm, 15 nm and 20 nm. The increase in Purcell factor as the gap is reduced is over an order of magnitude, but the relative peaks are smaller than for the nanorod. (**d**) As a consequence, the ratios ${{\rm{\Gamma }}}_{r}/{\rm{\Gamma }}$ for these nanostructures are slightly larger than for the nanorods. For both nanoparticles, smaller gaps are better for enhancing the far field detection of fluorescence, because the increase in the ratio ${{\rm{\Gamma }}}_{r}/{\rm{\Gamma }}$ as the gap is increased does not compensate for the large decrease in ${{\rm{\Gamma }}}_{r}$.

In applications to sensing, the greatest efficiency would be achieved by maximizing the number of photons radiated in the far field while minimizing the number of photons coupled to dark modes and absorbed, *i.e*. maximizing simultaneously ${{\rm{\Gamma }}}_{r}$ and ${{\rm{\Gamma }}}_{r}/{\rm{\Gamma }}$. In most cases, as the dipole gets closer to the nanoparticle, ${{\rm{\Gamma }}}_{r}$ increases while ${{\rm{\Gamma }}}_{r}/{\rm{\Gamma }}$ decreases and so one has to find the optimal compromise between maximizing these two quantities by examining how they vary as a function of the size of the gap. Purcell factors evaluated at the middle of the gap decrease by up to two orders of magnitude as the gap increases, as shown in Fig. 4(a) for gaps of 5 nm, 10 nm, 15 nm and 20 nm, and a nanorod with semi-axis of 60 nm. The corresponding variations in the ratios of ${{\rm{\Gamma }}}_{r}/{\rm{\Gamma }}$ are shown in Fig. 4(b) and indicate that for this type of nanostructure ${{\rm{\Gamma }}}_{r}/{\rm{\Gamma }}$ increases only by a few percent as the gap becomes wider. For a sphere with 60 nm radius, the increase in Purcell factor as the gap is reduced is comparable to that of the nanorod, see Fig. 4(c), but the variation of ${{\rm{\Gamma }}}_{r}/{\rm{\Gamma }}$ is slightly larger, Fig. 4(d). As the increase in the ratio ${{\rm{\Gamma }}}_{r}/{\rm{\Gamma }}$ does not compensate for the large decrease in the absolute values of ${{\rm{\Gamma }}}_{r}$ as the gap becomes larger, we conclude that smaller gaps are better for enhancing the far field detection of fluorescence for both types of nanoparticle. This is very different from what happens with small metallic nanospheres^22^ without a substrate, where ${{\rm{\Gamma }}}_{r}/{\rm{\Gamma }}$ is negligible for small gaps. From the point of view of applications, near bright resonances the limiting factor for the gap size may be due more to loading the emitters in the gap and limiting charge transfer than coupling to dark modes and absorption.

**Figure 5:**
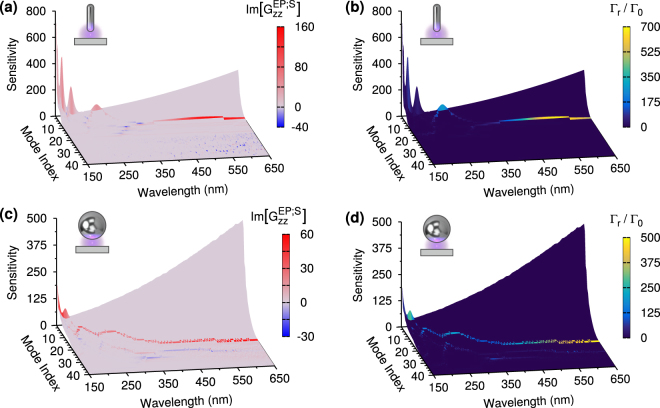
(**a**) Mode landscape for the nanorod with 60 nm semi- axis and 5 nm gap. The mode traces in these three dimensional plots are an intrinsic characteristic of the nanostructures and do not depend on the incident field. The vertical axis gives the sensitivity, whose maxima corresponds to mode resonances. The higher is the trace’s sensitivity, the higher is the corresponding mode amplitude for a given value of the coupling between the dipole field and the mode. The two horizontal axes are the wavelength and the mode number, which indicates the order of mode pairs according to the surface correlation between the internal and scattering modes of the pair. The mode traces are color coded according to the contributions to the Purcell factors shown. Note that the Purcell factors are dominated by a small number of modes and that for the peak at the longest wavelength the vertical variations of the mode trace are much smaller than for the other resonances. (**b**) Same mode landscape as in (**a**), but with mode traces color coded according to the far field radiative enhancement ${{\rm{\Gamma }}}_{r}/{{\rm{\Gamma }}}_{0}$. Only the two resonant modes at longer wavelengths contribute significantly to the radiative decay. Also for a nanosphere with 60 nm radius, only a small number of modes contribute significantly to the Purcell factor, as shown in (**c**), or the far field enhancement (**d**). The jitters of the bright modes in some landscapes are due to changes in mode indexes when several mode pairs have similar surface correlations.

The features observed in Figs 3 and 4 can be attributed to resonances of the principal modes, and the ability of those modes to radiate in the far field. Resonances correspond to maxima of the sensitivities of the modes and depend on the properties of the internal and external media, the geometry of the particles, and the size of the gap. Higher sensitivities correspond to larger mode amplitudes when an incident field couples to the modes, see methods. In Fig. 5 we show the mode landscapes, *i.e*. the traces of the mode sensitivities versus wavelength and mode number, for two of the nanostructures; the nanorod and nanosphere, with 60 nm semi-axis and radius, and with a 5 nm gap. The shape of the mode landscapes are independent of the incident field, and the mode indexes are arbitrarily assigned according to the surface correlation between mode pairs at each wavelength. With this format, significant resonances only appear towards the back of the landscapes, for modes with lower indexes, while modes with higher indexes are important only very close to the particle surface, and so we can limit the number of modes required in the following analysis.

In Fig. 5(a) we show the mode landscape for the nanorod, which is color coded according to the contribution of each mode to the Purcell factors via the expansion of the scattering Green’s function, Eq. (2), which appears in Eq. (1). Comparing the mode landscape with the Purcell factors shown in Fig. 4(a), we find that each peak is associated to one dominant mode. The mode traces shown in Fig. 5(b) are instead color coded according to the far field radiative enhancement, ${{\rm{\Gamma }}}_{r}/{{\rm{\Gamma }}}_{0}$, induced by each mode. These mode landscapes show that the radiative decay is also determined by a very low number of modes, but that only the two broader resonances at longer wavelengths correspond to strongly radiative bright modes, while the other resonances are due to dark modes which radiate more weakly in the far field.

Figures 5(c) and (d) show the mode landscape for the nanosphere color coded according to the contributions to the Purcell factors and the far field radiative enhancement, respectively. These landscapes show that, compared with the nanorod, the nanosphere has broader resonances with smaller peak values of the sensitivity; moreover, there are modes radiating in the far field for a wider interval of wavelengths, although less effectively than the nanorod’s bright modes. From the color coding of the traces, we also see that there are more modes coupled to the incident field, so that interference effects among these modes are more important than for the nanorod. The mode landscapes for the other particles – not included – show that as the semi-axis of the nanorod, or the radius of the nanosphere, is reduced, the resonances are blue shifted. However, the brightest modes are always the two modes with resonances at longer wavelengths for the nanorods, with the nanospheres having further weakly radiating modes resonant at shorter wavelengths.

Finally, by comparing the landscapes color coded with the Purcell factors, Figs 5(a) and (c), with those color coded with ${{\rm{\Gamma }}}_{r}/{{\rm{\Gamma }}}_{0}$, we see that the contribution of non resonant dark modes to the radiative resonances is negligible. This explains why the ratio ${{\rm{\Gamma }}}_{r}/{\rm{\Gamma }}$ shown in Fig. 4 does not change much as the gap is increased.

**Figure 6:**
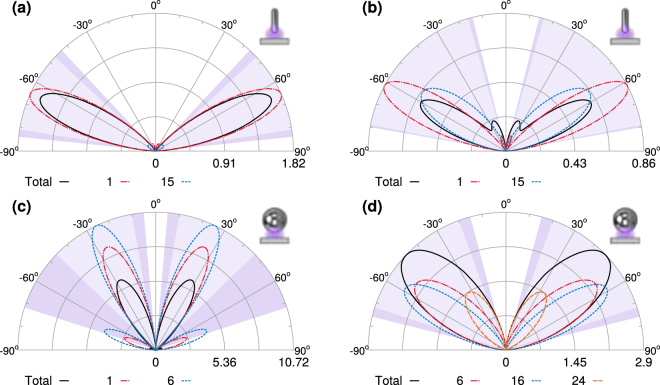
Radiation patterns for nanoparticles with a 60 nm semi-axis and 5 nm gap (black solid line) and the dominant modes at selected wavelengths. The light shaded areas indicate the angles into which 90% of the total energy is scattered, and the dark shaded areas increase this to 95%. For the nanorod there are two dominant modes (dashed red and blue lines) at 164 nm (**a**) and at 190 nm (**b**). The radiation pattern of the total field results from the interference among the modes; in both cases the total intensity is reduced along the direction of maximal emission of the dominant mode. For the nanosphere, the total field at the corresponding resonances is more strongly backscattered than for the nanorod, see (**c**) at 170 nm and (**d**) at 210 nm, where there is a third mode which contributes significantly to the total field.

**Figure 7:**
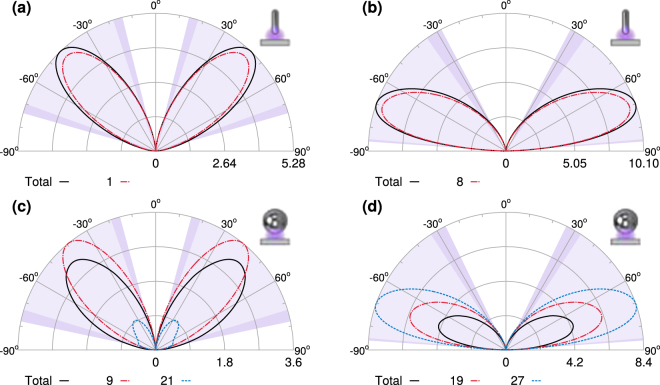
As in the previous figure, the light shaded areas indicate the angles into which 90% of the total energy is scattered, and the dark shaded areas increase this to 95%. For the nanorod the total field and the dominant mode radiation patterns are almost indistinguishable when there is only one dominant mode as at 260 nm, (**a**), and 560 nm, (**b**). In these two cases, the residual emission due to weak modes leads to a small increase along the direction of maximal emission of the dominant mode. The radiation patterns for the nanosphere at 280 nm (**c**) and 600 nm (**d**) strongly resemble those for the nanorod, however the total field for the nanosphere is always less than that of the resonant modes due to negative interference from another mode.

As we have shown, each spectral feature in Figs 3 and 4 can be described using a very small number of modes, and so we can further characterize these features by investigating the field distributions of the relevant modes in the near and far field. For the nanorod, with 60 nm semi-axis, there are two dominant modes which contribute significantly to the far field enhancement at the shorter wavelength resonances shown in Fig. 5(b), at 164 nm, shown in Fig. 6(a), and at 190 nm, Fig. 6(b). The radiation pattern of the total field results from the interference among the modes and in both cases the total intensity is reduced along the direction of maximal emission of the dominant mode. For the nanosphere, with 60 nm radius, the total fields at the corresponding resonances in Fig. 5(d) are more strongly backscattered than for the nanorod, shown in Fig. 6(c) at 170 nm, and in Fig. 6(d) at 210 nm, where there is a third mode which contributes significantly to the total field. For the nanorod, at the two bright resonances at longer wavelengths there is only one dominant mode and the total field and the dominant mode radiation patterns are almost indistinguishable, at 260 nm, Fig. 7(a), and 560 nm, Fig. 7(b). In these two cases, the residual emission due to weak modes leads to a small increase along the direction of maximal emission of the dominant mode. The radiation patterns at the longer wavelength resonances for the nanosphere at 280 nm, Fig. 7(c), and 600 nm, Fig. 7(d), strongly resemble those for the nanorod, however the total field for the nanosphere is always less than that of the resonant modes due to negative interference from another mode.

**Figure 8:**
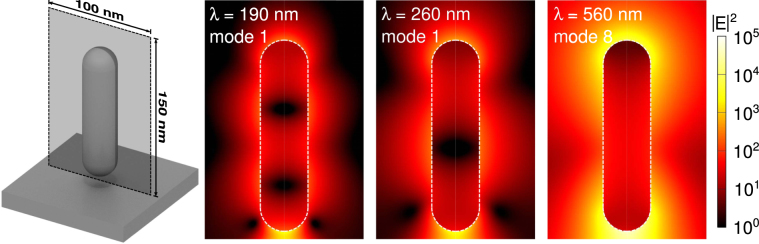
Near field intensity maps for individual mode pairs of the 60 nm nanorod with a 5 nm gap, corresponding to modes shown in Figs 6(a)and 7(a),(b). We show the logarithm of the electric field intensity, in arbitrary units, calculated on a 100 × 150 nm plane through the center of the particle, where the bottom edge is adjacent to the substrate interface. The particle boundary is indicated with a white dashed line. The bright mode resonant at 560 nm displays the lightning rod effect, and has a dipolar character. The number of observed nodes increases for resonant modes at shorter wavelengths, which have the character of higher order multipoles.

**Figure 9:**
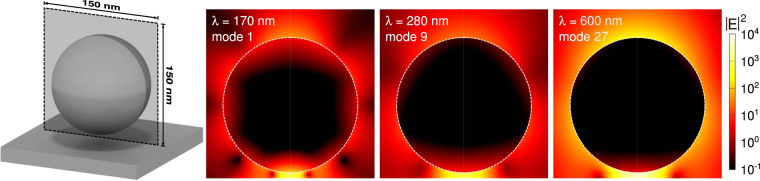
Near field intensity maps for individual mode pairs of the 60 nm nanosphere with a 5 nm gap, corresponding to modes shown in Figs 6(c) and 7(c),(d). We show the logarithm of the electric field intensity, in arbitrary units, calculated on a 150 × 150 nm plane through the center of the particle, where the bottom edge is adjacent to the substrate interface. The particle boundary is indicated with a white dashed line. We again observe an increase in the number of nodes in the intensity pattern for modes at shorter wavelengths.

In Figs 8 and 9, we show the near field intensity distributions for three of the brightest resonant modes at select wavelengths for the nanorod and nanosphere, respectively, and which correspond to modes shown in Figs 6 and 7. These field distributions were calculated on a plane normal to the substrate, with the bottom edge adjacent to the substrate interface, which bisects the particle. In both cases, the modes at longer wavelengths have a higher intensity and we observe that the number of nodes in the intensity patterns increases for modes at shorter wavelengths.

**Figure 10:**
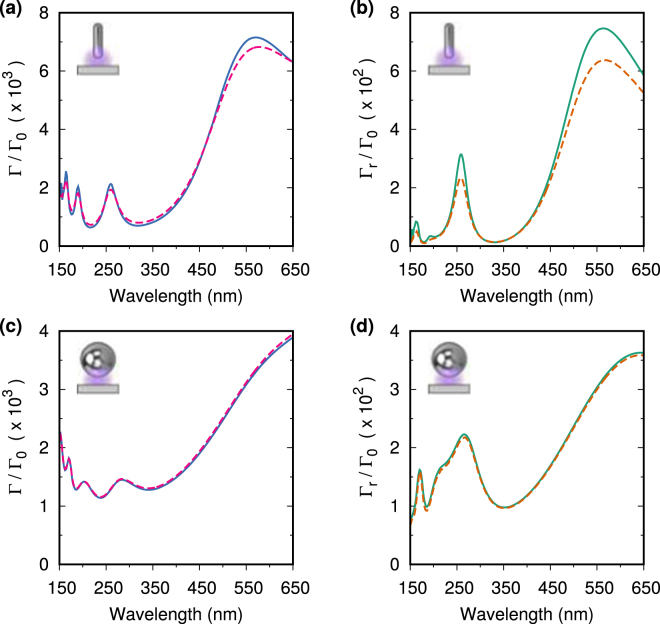
The Purcell factors in the middle of a 5 nm gap for a nanorod with 60 nm semi-axis (**a**), and a nanosphere with 60 nm radius (**c**), with (red dashed) and without (blue solid) the presence of surface roughness effects (*i.e*. with the damping amplitude *A* set to 1 and 0 respectively^35^). For the same particles, in (**b**) and (**d**) respectively, we show the far field radiative enhancement with (orange dashed) and without (green solid) the presence of surface roughness effects. Including surface roughness effects has little effect upon the nanosphere and reduces the magnitudes of the observed resonances of the nanorod by, at most, 25%.

Finally, it is not always easy in experiments to control the quality of the surface roughness of the metallic nanostructures. For this reason we compare, in Fig. 10, the Purcell factors and far field radiative enhancement of nanostructures with the same geometry, but different surface roughness. The main effect of surface roughness is a decrease in the scattering length of conduction electrons inside the metal, and can be accounted for by including an additional size dependent damping term in the dielectric function^35^. The presence of surface roughness affects the nanorod more strongly, as it has a dimension comparable to the mean free path of the conduction electrons in aluminum (≈20 nm^36^), but it only reduces the magnitudes of the observed resonances of the 60 nm nanorod by, at most, 25%. For the 60 nm nanosphere, the effect of surface roughness upon both the emission and far field radiation rates is negligible. As the presence of surface roughness does not affect strongly the results shown in this section, these very large enhancements can be observed in experiments.

## Conclusions

We have shown that the near and far field response of a gapped aluminum nanostructure made of a nanorod and a substrate is determined by very few pairs of principal internal and scattering modes. The two brightest modes have broad resonances that can be tuned from the visible to the ultraviolet by changing the length of the nanorods. In this way one can optimize the nanostructure with respect to the emitter simply choosing the size of the nanoparticle so that a radiative resonance is tuned with the fluorescence frequency. Because of the very low numbers of dominant modes near these bright resonances, the ratios ${{\rm{\Gamma }}}_{r}/{\rm{\Gamma }}$ depend only weakly on the size of the gap, so that the enhancement of the radiation rates can be maximized by using small gaps. An aluminum nanosphere coupled to a substrate has a similar response, with broader bright resonances and weaker enhancements. These resonances allows one to enhance by orders of magnitude the Purcell factors and the radiative rates from the visible to the deep ultraviolet.

The large enhancements found in this work, show that these nanostructures can be extremely useful to efficiently detect weakly fluorescent molecules and may open fluorescence up to metabolic biomarkers or amino acids which appear in the genetic code and for which fluorescence has not yet been detected. While experimental details such as the type of spacer used may be molecule dependent, the overall principles of gap enhanced fluorescence discussed in this paper should apply to a very broad class of emitters. Weakly radiative modes for nanorods, instead, have sharp line shapes and may be suitable for nanolasing in the ultraviolet, as they are for nanolasing in the visible^37^ and, depending on the dipole moment of the emitter, can be used to observe memory effects in the coupling between the emitter and radiation^17^.

## Methods

For the sake of completeness, we summarize here the main properties of the principal modes used in Eq. (2). We introduce a compact notation by defining six component (column) vectors for the electromagnetic fields, $F(x)={[{E}^{{\rm{T}}}(x),{H}^{{\rm{T}}}(x)]}^{{\rm{T}}}$, where T stands for transpose. The principal modes are found with the same procedure as for a particle in a homogeneous host medium^30^ and can also be used to study non-linear effects^38^. Central to this theory is the scalar product $f\cdot g\equiv {\sum }_{m}{\int }_{\partial {V}_{0}}{f}_{m}^{\ast }(a){g}_{m}(a)da$, where the lower cases are used to indicate the projections of the six dimensional electromagnetic fields on the surface of the particle $\partial {V}_{0}$, the index *m* labels the four tangent components (two electric and two magnetic), and the asterisk indicates the complex conjugate. This scalar product is a measure of the spatial correlation between fields over $\partial {V}_{0}$ and can be used to define projections.

The most important features of scattering and internal principal modes, $\{{s}_{n}\}$ and $\{{i}_{n}\}$, are that they are correlated pairwise as Mie’s modes of spheres and their correlations ${s}_{n}\cdot {i}_{n}$ are invariant properties^30^ that depend only upon the permittivity and permeability of the media, the shape and dimension of the particle, and the size of the gap. In applying this theory to nanospheres near a substrate, the internal principal modes are combinations of the internal Mie modes, while the principal scattering modes are combinations of radiating electric and magnetic dipoles, distributed inside the nanosphere, together with their reflections from the substrate^29,39^. For nanorods, both the internal and the scattering principal modes are combinations of electric and magnetic dipoles distributed inside the nanorod, with the scattering modes including also reflected terms. In this way the multiple scattering between the particle and the substrate is calculated to all orders and affects both the position of the mode resonances and the way modes couple to and propagate light^32^.

Given the field ${F}^{0}={[{[{G}_{0}^{EP}(x,x^{\prime} ){\bf{p}}(x^{\prime} )]}^{{\rm{T}}},[-i{(\mu \omega )}^{-1}\nabla \times {G}_{0}^{EP}(x,x^{\prime} ){\bf{p}}(x^{\prime} {)]}^{{\rm{T}}}]}^{{\rm{T}}}$ emitted by the dipole and incident on the particle, the mode amplitudes are

3 $${a}_{n}^{i}({\bf{p}}(x^{\prime} ))={i^{\prime} }_{n}\cdot {F}^{0}\mathrm{/[1}-{({s}_{n}\cdot {i}_{n})}^{2}],$$

4 $${a}_{n}^{s}({\bf{p}}(x^{\prime} ))=-{s^{\prime} }_{n}\cdot {F}^{0}\mathrm{/[1}-{({s}_{n}\cdot {i}_{n})}^{2}],$$

with ${i^{\prime} }_{n}\equiv {i}_{n}-({s}_{n}\cdot {i}_{n}){s}_{n}$ and ${s^{\prime} }_{n}\equiv {s}_{n}-({s}_{n}\cdot {i}_{n}){i}_{n}$. The principal modes are normalized so that ${i}_{n}\cdot {i}_{n}={s}_{n}\cdot {s}_{n}=1$, $0\le {i}_{n}\cdot {s}_{n}={s}_{n}\cdot {i}_{n}=\le 1$, ${i^{\prime} }_{n}$ is orthogonal to ${s}_{n}$, ${s^{\prime} }_{n}$ is orthogonal to *i*_*n*_ and ${i^{\prime} }_{n}\cdot {i}_{n}={s^{\prime} }_{n}\cdot {s}_{n}=1-{({s}_{n}\cdot {i}_{n})}^{2}$. Note that the amplitudes of the modes depend on the sensitivity, ${\mathrm{[1}-{({s}_{n}\cdot {i}_{n})}^{2}]}^{-1}$, which is an intrinsic property of the modes and does not depend on the incident field, and on the spatial distribution of the incident field on the surface of the particle through the terms ${i^{\prime} }_{n}\cdot {F}^{0}$ and ${s^{\prime} }_{n}\cdot {F}^{0}$.

**Figure 11:**
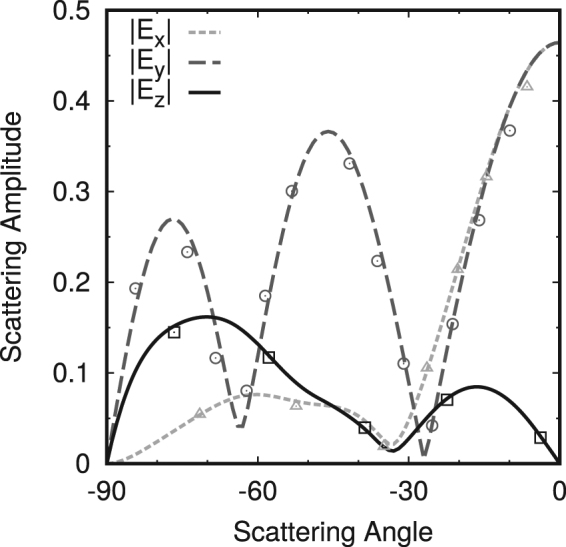
Numerical verification of the code used in this paper. Back scattered electric field component amplitudes for a 760 nm diameter polystyrene sphere (refractive index *n* = 1.59) on a silicon substrate (refractive index *n* = 3.8), excited by a plane wave at normal incidence (*λ* = 632.8 nm). Points show data from literature^41^.

In the numerical simulations shown in this work we have used between 100 and 150 principal modes, so that the fractional surface error, $|{f}^{0}-{f}^{I}+{f}^{S}{|}^{2}/|{f}^{0}{|}^{2}$ ^40^, was of the order of 10^−2^ or less. Here ${f}^{I}$ and ${f}^{S}$ are the projections, on the surface of the particle, of the scattered and internal fields, respectively. A validation of the method is given in Fig. 11 where calculations for a polystyrene sphere on a silica substrate^41^ have been reproduced.
